# Translational Studies of Alcoholism: Bridging the Gap

**Published:** 2008

**Authors:** Natalie M. Zahr, Edith V. Sullivan

**Keywords:** Alcohol dependence, alcoholism, chronic alcohol exposure, alcohol and other drug effects and consequences, genetic factors, environmental factors, brain, neurobiology, translational studies, human studies, animal studies, animal models

## Abstract

Human studies are necessary to identify and classify the brain systems predisposing individuals to develop alcohol use disorders and those modified by alcohol, while animal models of alcoholism are essential for a mechanistic understanding of how chronic voluntary alcohol consumption becomes compulsive, how brain systems become damaged, and how damage resolves. Our current knowledge of the neuroscience of alcohol dependence has evolved from the interchange of information gathered from both human alcoholics and animal models of alcoholism. Together, studies in humans and animal models have provided support for the involvement of specific brain structures over the course of alcohol addiction, including the prefrontal cortex, basal ganglia, cerebellum, amygdala, hippocampus, and the hypothalamic–pituitary–adrenal axis.

What currently is known about alcohol’s effects on the brain has benefited from translational research—the parallel study of humans with alcohol dependence and of animal models that mimic targeted aspects of this complex disease. Human studies provide a full depiction of the consequences of chronic alcohol exposure, but they are limited by ethical considerations for experimentation of rigorous controls of relevant variables. Animal models, on the other hand, can distinguish components of the addiction processes but cannot fully represent the human condition.

In humans, 40 to 60 percent of the risk for alcoholism can be attributed to genetic factors. These genetic factors interact with environmental factors (e.g., early-life stress, family structure, peer pressure, or the social environment; McKenzie et al. 2005) to influence an individual’s vulnerability to alcohol problems (Prescott and Kendler 1999). The genetic component has been modeled by breeding animal strains (predominantly rats and mice) with a high preference for alcohol (e.g., the alcohol preferring [P] and nonpreferring [NP] rats, high-alcohol–drinking [HAD] and low-alcohol–drinking [LAD] rats, the high-alcohol–preferring [HAP] mouse, and C57 black mice). The environment also has been modeled, for example, by separating young monkeys from their mothers, which reproduces early-life stress (Barr et al. 2004).

The last quarter century has seen a plethora of technologies capable of exploring the human animal in vivo, and many have been applied to alcohol-related research. Currently available noninvasive human technologies (reviewed elsewhere in this two-part series) include electroencephalogram (EEG) (Rangaswamy and Porjesz, pp. 238–242), functional magnetic resonance imaging (fMRI) (Nagel and Kroenke, pp. 243–246; Rosenbloom and Pfefferbaum, Part 2), magnetic resonance spectroscopy (MR spectroscopy) (Nagel and Kroenke, pp. 243–246), single- photon emission computed tomography (SPECT) (e.g., Abi-Dargham et al. 1998), and positron emission tomography (PET) (Thanos et al., pp. 233–237). Further investigation of alcohol’s effects at the cellular (e.g., He and Crews 2008; Tupala and Tiihonen 2004), molecular (e.g., Alexander-Kaufman et al. 2007), and genetic (e.g., Dodd et al. 2006; Saba et al., pp. 272–274) levels is made possible by carefully screened human postmortem brain tissue (Harper et al. 2003*a*).

Even with these new technologies, animal models continue to have a vital role, enabling researchers to better interpret the implications of new findings. Moreover, the wide variation (or heterogeneity) of alcoholic populations examined with respect to genetic predisposition, age of onset, pattern of drinking, frequency of withdrawals, length of sobriety, nutritional, and hepatic status has hampered researchers’ attempts to isolate only those specific brain regions affected by alcohol per se. This heterogeneity, and the complexity that it introduces, makes it difficult to thoroughly characterize the disorder (see Oscar-Berman 2000). Animal models, in contrast to the indefinite natural course of alcohol use in humans, allow researchers to determine alcohol toxicity in a way that allows them to control for multiple genetic, environmental, and alcohol consumption factors.

Alcohol dependence is defined in the *Diagnostic and Statistical Manual, Fourth Edition* (DSM–IV) as the presence of three of a total of seven possible criteria within a 12-month period (figure 1A; American Psychiatric Association 1994). The diagnosis of alcohol abuse with DSM–IV criteria has helped standardize the classification of alcoholics, both across national and international research facilities and time (Harper et al. 2003*b*).

In modeling alcoholism, a series of conditions that attempt to parallel DSM–IV criteria have been established (figure 1B; Cicero et al. 1971). Of the currently available animal models, the monkey (e.g., *Macaca fascicularis*) and the P rat best fulfill these criteria. The nonhuman primate is particularly suitable, as it has genetic, neuroanatomical, behavioral, and social similarities with humans (Premack 2007). Furthermore, in contrast to other species (notably the wild-type rat), monkeys will self-administer alcohol (Grant et al. 2008). The P rat was developed from a Wistar foundational stock in Indiana and is in its 65th generation for selection of alcohol preference. The P rat is well-characterized behaviorally and neurobiologically (Li et al. 1993; McBride and Li 1998) and satisfies the criteria proposed as essential for an animal model of alcoholism (Cicero et al. 1971).

The goal of this review is to identify key findings in humans, highlighting current theories regarding the brain systems involved in alcoholism, and to examine the currently available animal models of alcoholism within the context of those theories. What should emerge is that (1) human studies are necessary to identify and classify the brain systems predisposing individuals to develop alcohol use disorders and those modified by alcohol; (2) animal models of alcoholism are essential for a mechanistic understanding of how chronic voluntary alcohol consumption becomes compulsive, how brain systems become damaged, and how damage resolves; and (3) human studies then must create methods for testing target mechanisms of alcohol dependency identified in rigorous animal studies.

## Theories of Alcoholism Derived from Human and Animal Studies

Neurobiological theories of alcoholism offer a framework from which to develop, design, and test hypothesis-driven experiments in human alcoholics and animal models of alcoholism. Here, we present exemplary theories derived from these studies. These theories involve mechanisms of disinhibition, reward, habit formation, stress, and inflammation and have implications for recovery. Findings from animal models that have either helped in the development or aided in the support of these theories as they inform our understanding of the mechanisms of human alcoholism are highlighted (see chapter 9; Koob and Le Moal 2006).

### Disinhibition

About one-half to two-thirds of alcohol-dependent individuals develop mild-tomoderate deficits in complex cognitive and motor processes. The skills typically affected are related to executive functioning, a multicomponent, higher-order cognitive construct involved in the self-regulation of goal-directed behavior. Deficits in executive functioning are associated with tasks related to working memory, problem solving, temporal ordering, and response inhibition (see Fein et al. 1990; Oscar-Berman and Marinkovic 2007 for reviews of behaviors modified by alcoholism; Sullivan et al. 2000*b*).

The class of behaviors associated with executive dysfunction has a common psychological mechanism—disinhibition, which describes the behavior of individuals who exhibit a limited capacity to edit or manage their immediate impulsive response to a situation or are poorly motivated to do so (e.g., DSM–IV criteria 3, inability to control alcohol use, Fein et al. 1990; Finn et al. 1992; Oscar-Berman and Hunter 1993; Parsons 1993; Sinha et al. 1989; Sullivan et al. 2003). Alcoholics lacking impulse control also tend to exhibit novelty-seeking, aggressive, and antisocial behaviors and are sometimes referred to as type II alcoholics (Cloninger et al. 1985). When monkeys are separated from their mothers at birth for 6 months, they demonstrate behaviors such as infrequent social interactions, less competent social behaviors, and higher alcohol consumption rates compared with their mother-reared peers (Higley et al. 1996). These behaviors generally are consistent with the type II alcoholic personality. In rodents, disinhibition has been quantified using the plus-maze test, which draws on the animals’ aversion to open areas and their desire to explore novel environments. Mice administered alcohol spend more time in open areas than mice not exposed (Durcan and Lister 1988). Heightened exploration of novel environments is evidence of disinhibition.

**Figure 2:**
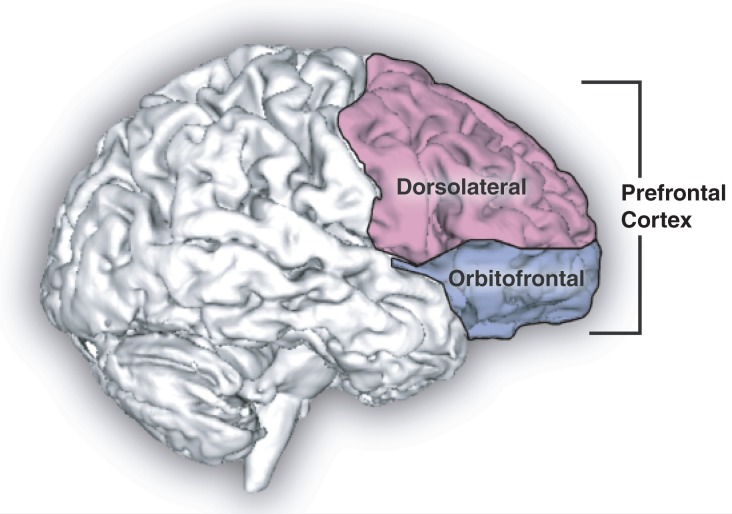
Sagittal human brain with cortical regions delineated.

Executive dysfunction is associated with damage to the dorsolateral prefrontal cortex and its subcortical connections, whereas disinhibited behavior is linked to the orbitofrontal cortex and its circuitry (Cummings 1995) (figure 2).

Postmortem examination of brain tissue of human alcoholics without co-occurring complications that could alter results demonstrates a decreased number of neurons in the superior frontal cortex compared with control subjects (Kril et al. 1997). Furthermore, deficits in regional tissue volume, especially prevalent in the prefrontal cortex of similar alcoholics (Pfefferbaum et al. 1992), have been quantified using various anatomical MRI methods (reviewed by Adalsteinsson et al. 2002; Sullivan and Pfefferbaum 2008). However, little evidence exists that shows frontal tissue damage in animal models of alcoholism. For example, postmortem evaluation of the canine brain after 1 year of alcohol exposure did not reveal statistically significant differences in frontal cortical thickness or neuron population compared with unexposed animals (Hansen et al. 1991). In the rat, neuronal damage has been observed in several cortical regions (e.g., entorhinal, insular, piriform, and perirhinal cortices) after administration of alcohol in a pattern reflective of binge drinking (i.e., delivery of alcohol three times daily for 4 days; Collins et al. 1996), but neuronal loss in the frontal association cortex has been reported only when the alcohol exposure protocol included bouts of thiamine deficiency (Kril and Homewood 1993).

In summary, alcoholics appear to have either innate or acquired behaviors characterized psychologically as disinhibition, and this characteristic is shared by monkeys and rodents exposed to alcohol. However, only humans show evidence of tissue shrinkage as well as atrophy in prefrontal cortical regions as a consequence of chronic alcohol exposure. Despite the absence of evidence for prefrontal damage in animal models of alcoholism, they have been indispensable in helping to distinguish the mechanisms underlying alcohol’s effects on these prefrontal regions.

**Figure 3:**
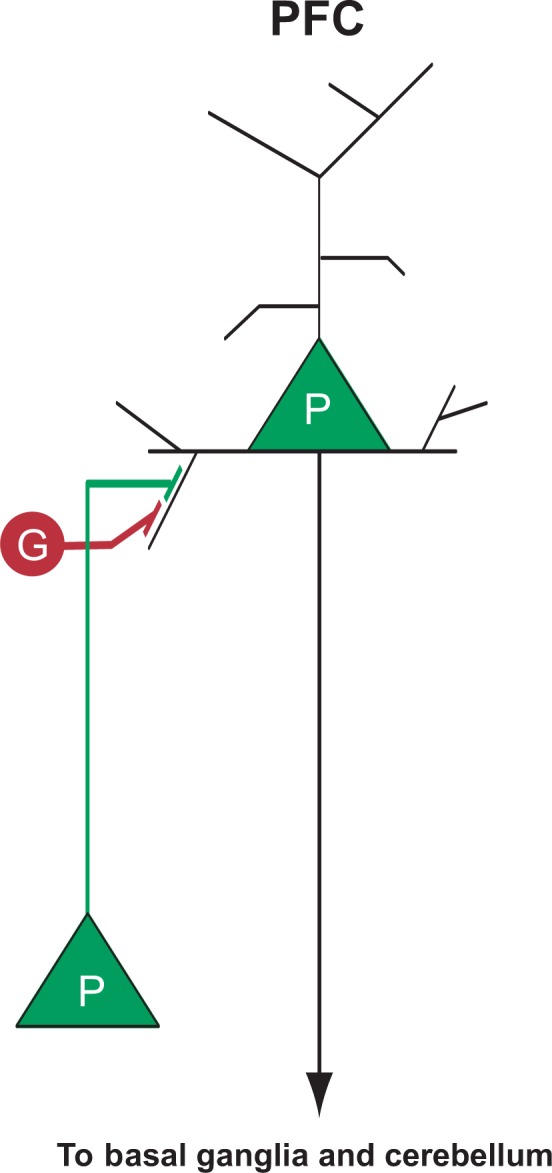
Simplified schematic of excitatory (other pyramidal [P] neurons) and inhibitory (GABAergic interneurons [G]) input to a pyramidal neuron in the prefrontal cortex (PFC).

The prefrontal cortex is fundamentally composed of functional modules of excitatory pyramidal projection neurons and inhibitory (γ-aminobutyric acid [GABA]) interneurons. The processing of information within these local circuits is critically dependent on GABA acting on GABA_A_ receptors (Krimer and Goldman-Rakic 2001; Ticku and Mehta 1990); (figure 3). A feline model was the first to provide evidence that alcohol modifies GABA_A_ receptor function. Using extracellular single-unit recordings in the precruciate cortex of anesthetized cats, it was found that alcohol (given at doses associated with human intoxication) rapidly and reversibly enhanced GABA_A_ and its receptor activity, thus creating an overall inhibitory effect. This enhancement was specific to GABA, as the effect was not observed with glycine, dopamine, or serotonin (Nestoros 1980).

Human electrophysiology research also contributed to discerning the role GABA plays in response to alcohol exposure. The β wave, typically observed in normal waking consciousness, describes brain activity greater than 12 Hz that arises from frontal brain regions and is generated by inhibitory interneurons (Whittington et al. 2000). The β wave is accentuated and rhythmic in the resting EEG of alcoholics and children of alcoholics (Porjesz and Rangaswamy 2007). Collaborative Studies on the Genetics of Alcoholism (COGA) researchers recently identified a significant linkage between the β wave and a GABA_A_ receptor gene in alcoholic individuals (Porjesz et al. 2002). Taken together, these findings have led to the hypothesis that subtle alterations in the structure or function of GABA_A_ receptors may disrupt local cortical processing and information the cortex relays to other brain regions, thereby contributing to the deficits in executive function seen in alcoholism (Agrawal et al. 2006). More research is needed to determine whether altered GABA_A_ receptors in the prefrontal cortex underlie the deficits in executive control of behavior observed in alcoholics. Nevertheless, the associations between a GABA_A_ receptor variant, the β wave, and disinhibited behavior in alcoholics clearly demonstrates the unique relationship between the brain’s structure and function, and animal models have been vital in helping to better understand this relationship.

#### Limitations of Animal Models of Disinhibition

The nonhuman primate is an especially appropriate model for studying disinhibition at the behavioral and frontal brain level because the size of the monkey’s cerebral cortex is similar to that seen in humans (Grant and Bennett 2003). Other animal models, however, do not correspond as well.

For example, postmortem studies in rats suggest that the distributions of GABA_A_ receptors differs from that of humans (Richards et al. 1987). This could have significant implications. A distinct distribution of receptors or differing subunit expression across species could lead to variations in the brain’s function at the molecular, cellular, and electrophysiological levels. For example, the P300, the most robust feature of event-related potentials (i.e., electrophysiological responses to stimuli with characteristic waveforms) (see Rangaswamy and Porjesz, pp. 238–242), manifested in response to unpredictable stimuli (Kaufmann et al. 1982) and emanating partially from the frontal cortex, is reduced in alcoholics (Begleiter et al. 1984; Johnson et al. 1984; Polich et al. 1994). Yet, in a recently developed mouse model of high alcohol consumption, the high-alcohol–preferring animals had an increased P3 latency when compared with the low-alcohol–preferring mice (Slawecki et al. 2003).

### Frontocerebellar Circuitry

Despite evidence for compromised executive function and volume deficits in the frontal lobes of alcoholics, few instances have shown that frontal abnormalities predict impaired executive function (Adams et al. 1995; Cardenas et al. 2007; Dao-Castellana et al. 1998; Rosse et al. 1997). This has spawned theories that there must be alternative or additional areas of brain disruption associated with alcoholism.

In one study, dogs that were given alcohol at levels which mimicked intoxication in humans (i.e., the dogs achieved a blood alcohol level [BAL] of 231 ± 18 mg/dl) showed a general decline in brain blood flow measured with tracer microspheres. The decline was most marked and persistent in the cerebellum (Friedman et al. 1984), an area of the brain that is particularly vulnerable to damage from excessive alcohol exposure. Indeed, postmortem studies support this finding, showing neuronal loss (Baker et al. 1999; Harper 1998; Phillips et al. 1987; Torvik and Torp 1986) and cellular dysmorphology (Andersen 2004; Victor et al. 1959) in the cerebellum of alcoholics. MRI also reveals significant volume deficits of the cerebellum of alcoholics that are especially profound in the anterior superior vermis (Andersen 2004; Sullivan et al. 2000*a*). These findings also are evident in animal models. Lower neuronal counts (Tavares and Paula-Barbosa 1982) and cellular dysmorphology (Dlugos and Pentney 1997; Pentney et al. 1989) have been observed in the cerebellum of the rat brain chronically exposed to alcohol. MRI of the P rat with moderate BALs of 125 mg/dl also demonstrated modifications in the cerebellum (Pfefferbaum et al. 2006*a*).

**Figure 4:**
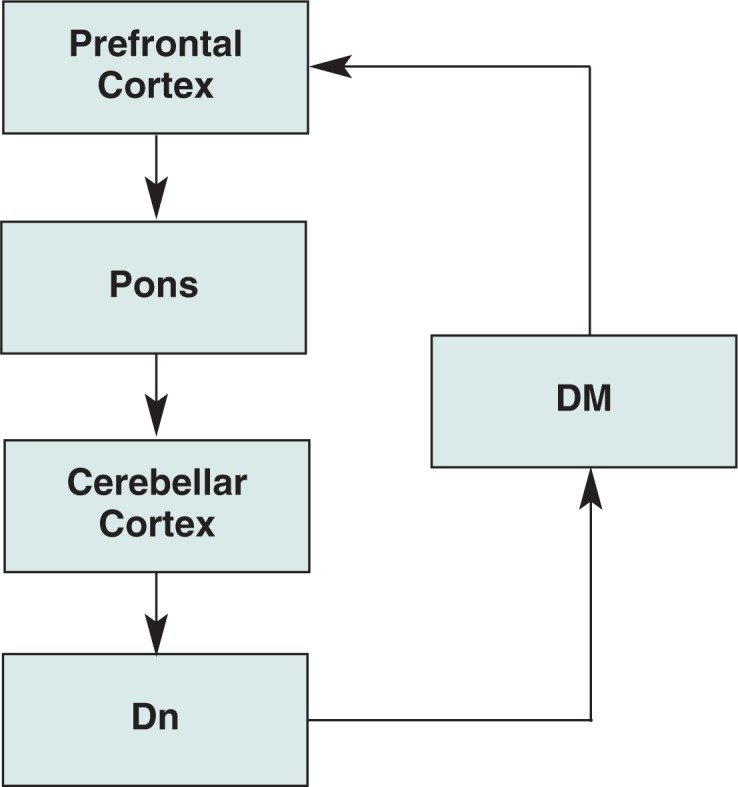
Simplified schematic of frontocerebellar circuitry. NOTES: DM = dorsomedial nucleus of the thalamus; Dn = dentate nucleus.

The importance of these findings is far-reaching. The cerebellum now is recognized to contribute significantly to functions classically associated with the frontal lobes, including verbal associative learning, word production, problem solving, cognitive planning, attentional set shifting, and working memory (Courchesne et al. 1994; Schmahmann 2000). Our updated understanding of cerebellar function has been supported by anatomical evidence in the monkey (*Cebus apella*) that cerebellar projections extend as far as area 46 (roughly corresponding with the dorsolateral prefrontal cortex, Kelly and Strick 2003; figure 4), suggesting the presence of a pathway whereby the cerebellum may access executive functions.

In alcoholics, certain regions of cerebellar volume shrinkage are better predictors of executive impairment than frontal lobe volumes (Sullivan et al. 2003). Still, the relationship between the degree of cerebellar damage and cognitive functioning in alcoholics has not been unequivocally established (Davila et al. 1994; Johnson-Greene et al. 1997), and the theory that frontocerebellar degradation contributes to the cognitive sequelae of alcoholism warrants further investigation (Fitzpatrick et al. 2008).

#### Limitations of Animal Models of Frontocerebellar Circuitry

As with other brain structures, the cerebellum as a whole is disproportionately enlarged in humans and nonhuman primates compared with lower species (Semendeferi and Damasio 2000; Sultan and Braitenberg 1993), and its volume of white matter is exponentially greater in more (phylogenetically) recent species (Bush and Allman 2003). The organization of cerebellar inputs from the cortex via the pons (i.e., mossy fibers) is significantly different in humans than in rats (Paula-Barbosa and Sobrinho-Simoes 1976). Cerebellar activation of cortical regions also has been shown to differ among the rat, cat, and monkey (Tolbert et al. 1978; Yamamoto et al. 2004). In addition, the GABA_A_ receptor distribution in the cerebellum has been found to be different between humans and rats (Kume and Albin 1994). The distribution of dopamine receptors in the cerebellum also differs between the mouse, rat, guinea pig, cat, and monkey (Camps et al. 1990). Finally, the pattern of cerebellar pathology in response to alcohol in rodents is markedly different from that observed in humans (Tavares et al. 1987). Such ubiquitous evidence for structural differences in the cerebellum among various species has implications for function and suggests that the study of frontocerebellar circuitry disruption in alcoholism may be difficult in animal models.

### Reward

One of the original theories of alcohol abuse was that alcohol is consumed for its rewarding (e.g., antianxiety) properties. A reward reinforces behavior; positive reinforcement describes a situation in which a rewarding stimulus (i.e., alcohol) increases the probability of (and motivation for) an appetitive instrumental response (i.e., alcohol seeking; discussed in this issue and in Part 2).

**Figure 5:**
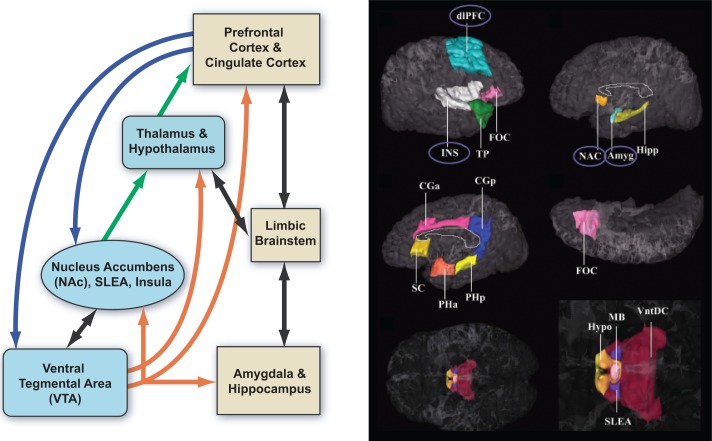
**Left panel)** Extended reward and oversight system. **Right panel)** Cortical and subcortical regions in the “reward network” in which alcoholics have smaller volumes (covaried for age and total cerebral volume). Smaller volumes are circled. NOTE: Amyg = amygdala; CGa, CGp = cingulate (anterior, posterior); dlPFC = dorsolateral prefrontal cortex; FOC = orbitofrontal cortex; Hipp = hippocampus; Hypo = hypothalamus; INS = insula; MB = mammillary bodies; NAC = nucleus accmbens; PHa, PHp = parahippocampal gyrus (ant, post); SC = subcallosal cortex; SLEA = sublentic-ular extended amygdala; TP = temporal pole; VntDC = ventral diencephalon. SOURCE: Reprinted from *Biological Psychiatry,* Vol. 64, No. 3, Makris, N.; Oscar-Berman, M; Jaffin, S.K.; Hodge, S.M.; et al. Decreased volume of the brain reward system in alcoholism. Copyright 2008, with permission from Elsevier.

A large body of research on alcohol addiction has focused on the mesolimbic dopaminergic system (e.g., Brodie et al. 1990), with dopamine neurons in the ventral tegmental area (VTA) and their targets in the ventral striatum (i.e., nucleus accumbens) playing a key role in this circuitry inextricably linked to the concept of reward (figure 5, left panel).

An increase in dopamine release in the nucleus accumbens is associated with the presence of a rewarding stimulus such as food (Blum et al. 2000), but release may be three- to fivefold higher in response to alcohol, at least in acute stages (Di Chiara and Imperato 1988; Wise 2002). In humans, various methods have confirmed that key elements of the reward circuit are activated during initial alcohol use and the early binge/intoxication stage. Long-term alcohol exposure reduces the volume of key basal ganglia structures, including the dorsolateral prefrontal cortex, insula, nucleus accumbens, and amygdala (Makris et al. 2008) (figure 5, right panel). In the P rat, 8 weeks of exposure to free-choice alcohol resulted in changes in basal ganglia structures (i.e., caudate, putamen, nucleus accumbens, globus pallidus, substantia nigra, and ventral tegmental area) (Sable et al. 2005).

Classically, a major impediment to the reward theory of alcoholism has been that unlike cocaine or amphetamine, agents that act directly on the dopamine transporter to increase dopamine release, no direct effect of alcohol on dopamine neurons could be demonstrated. Now, various results from animal studies have converged to provide a potential mechanism for alcohol-induced dopamine release. When μ-opioid receptors in the VTA of wild-type Sprague-Dawley rats are activated, there is an increase in dopamine release (measured with in vivo microdialysis) in the nucleus accumbens (Spanagel et al. 1992). Indeed, μ-opioid receptor activation hyperpolarizes (i.e., suppresses or inactivates) GABAergic interneurons in the VTA, thereby releasing dopaminergic neurons from spontaneous inhibition (Johnson and North 1992) and facilitating dopamine release (Di Chiara and North 1992; Margolis et al. 2003).

Increased dopamine release also has been measured using an electrophysiological technique known as patch-clamp recording. Studies using midbrain slices from the rat showed that alcohol, by activating μ-opioid receptors localized on GABAergic interneurons of the VTA, inhibits GABAergic transmission, thereby facilitating dopamine cell firing and enhancing dopamine release in the nucleus accumbens (Xiao et al. 2007). This mechanism of action was further substantiated by evidence that alcohol- stimulated dopamine release is decreased in mice in which the μ-opioid receptor is genetically altered (or knocked out) (Job et al. 2007). This is particularly relevant to the human condition because researchers speculate that innate differences in dopamine neurotransmission may predispose individuals to excessive alcohol consumption (see Cowen and Lawrence 1999).

#### Limitations of Animal Models of Reward

Humans and rodents react differently to pharmacological agents that target dopamine receptors located both locally in the VTA and distally in the striatum and prefrontal cortex (Wood et al. 2006). Even within a species, strains may have different dopamine receptor binding properties and distributions (Yaroslavsky et al. 2006; Zamudio et al. 2005); more “effective” receptors may be associated with innate deficits in dopamine levels. The subregional topography of the dopamine transporter, responsible for dopamine uptake after its release, also has been shown to be inconsistent across species (e.g., rodent, monkey, and human) (Smith and Porrino 2008), a finding that also may have a significant impact on extracellular dopamine levels and innate responses to rewarding stimuli.

### Habit Formation

At some point between initial exposure and dependence, the consumption of alcohol seems to proceed automatically, as a habitual response to antecedent stimuli. This transition may be the result of a complex interchange between executive and habit systems (Redish et al. 2008). Habitual drinking behavior becomes difficult to break using cognitive mechanisms because of an underperformance of executive systems (Jentsch and Taylor 1999), an overperformance of habit systems (Robbins and Everitt 1999), or because of an imbalance between the two systems (Bechara 2005).

Although not explored comprehensively, brain systems potentially contributing to habit formation include the striatum, cerebellum, amygdala, and, in limited conditions (e.g., trace conditioning; see below for more information), the hippocampus. Indeed, any system involved in “automatic” or implicit learning (i.e., learning without awareness) is fundamental for the establishment of habits (for reviews, Eichenbaum and Cohen 2001). Recent work in rodents has focused on the contribution of the corticostriatal network to habit formation. This work suggests that a switch occurs in the control of instrumental behavior so that the associative or medial striatum, important in the early, goal-directed stage of action, is overridden by the sensorimotor or lateral striatum at the later, more habitual stage (reviewed by Yin, Part 2). Furthermore, several types of classical conditioning/implicit learning paradigms, including eye-blink conditioning (McGlinchy-Berroth et al. 1994), visual discrimination learning (Rogers et al. 2000), and contextual cue discrimination learning (Greene et al. 2007), have been shown in both animal and human studies to be critically dependent on selective cerebellar sites.

The amygdala is another brain structure implicated in habit formation. It plays a role in emotional regulation and behavioral control (for review, see McBride 2002). It has been connected to a specific type of conditioned learning—Pavlovian fear conditioning (Volkow et al. 2002)—in which a neutral conditioned stimulus is paired with a fear-inducing unconditioned stimulus, so that animals come to exhibit a conditioned fear response to the conditioned stimulus. Extensive evidence indicates that the basolateral amgydala is critical for experimental extinction of this acquired fear (Akirav and Maroun 2007).

Although there is support for (Alvarez et al. 1989) and against (Kril et al. 1997) neuronal loss in the amgdala of chronic alcoholics, several in vivo MRI studies provide evidence for volume deficits in the amygdala of abstinent, long-term chronic alcoholics (Fein et al. 2006; Makris et al. 2008). Furthermore, modifications of the GABA_A_ receptor in the basolateral amygdala have been reported in Cynomolgus macaques exposed to alcohol for 18 months (Anderson et al. 2007). How altered GABA receptor function, loss of neurons, or volume reductions in the amygdala contribute to the formation of an alcohol habit remains to be seen.

In another specific form of classical conditioning—termed trace conditioning—a silent period elapses between the occurrence of the conditioned stimulus and the delivery of the unconditioned stimulus (i.e., the conditioned stimulus and unconditioned stimulus are not paired at precisely the same moment, but rather, there is a silent period between the presentation of the conditioned stimulus and unconditioned stimulus).

Evidence from animal (Weible et al. 2006) and human (Cheng et al. 2008) research suggests that the hippocampus plays a critical role during trace eye-blink conditioning. MRI provides in vivo evidence for volume deficits in the anterior hippocampus of chronic alcoholic individuals (Agartz et al. 1999; Sullivan et al. 1995). However, other than its effect on volume shrinkage, alcohol does not appear to have an effect on the number of hippocampal neurons, per se, as shown in studies using postmortem human hippocampal tissue (Harding et al. 1997; Kril et al. 1997). In contrast to the human condition, chronic exposure to alcohol in rodents induces a decrease in neuronal counts in CA1 to CA4 regions of the hippocampus in female Sprague-Dawley (Bengoechea and Gonzalo 1991) and male Long-Evans (Walker et al. 1980) rats and a decrease in the number of pyramidal neurons in CA1 and CA2 regions of the hippocampus of mice (Pawlak et al. 2002). Compared with humans, rodents have a disproportionately larger hippocampal volume, which may account for the notable differences in neuronal loss observed between humans and rodents. Nonetheless, modified hippocampal anatomy may contribute to impaired trace eye-blink conditioning in rats exposed to a binge-like patterns of alcohol in the early postnatal period (Tran et al. 2007) and in nonamnesic alcoholic individuals (McGlinchey et al. 2005).

In humans, both the striatum and the cerebellum have been shown to participate in the automatization process during the late learning stage of a repeated visuomotor sequence (Doyon et al. 1997) and of a sequence of finger movements (Doyon et al. 1998). Yet the collaborative contributions of the striatum, cerebellum, amygdala, and hippocampus to the formation of an alcohol consumption habit have yet to be demonstrated.

### Stress

**Figure 6:**
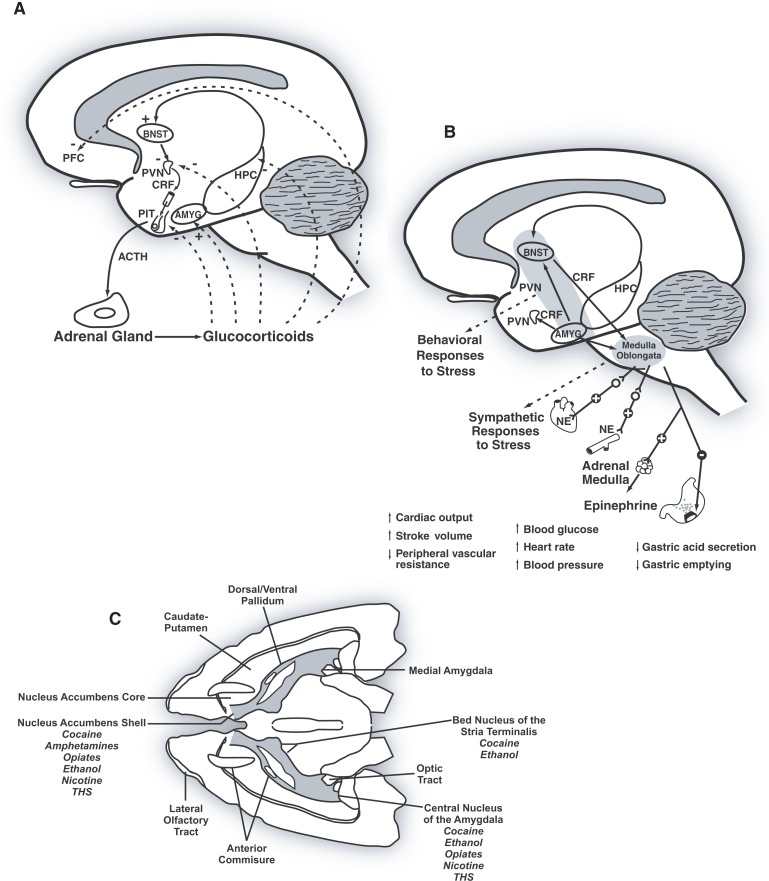
**A)** Human hypothalamic–pituitary–adrenal brain stress system. **B)** Human extrahypothalamic cortiocotropin–releasing factor (CRF) brain stress system. **C)** Rodent extrahypothalamic CRF brain stress system. SOURCE: A and B from Koob, G.F., and Le Moal, M. Drug Addiction, Dysregulation of Reward, and Allostasis. *Neuropsychopharmacology* 24:97–129, 2001.; C from Koob G.F., Alcoholism: Allostasis and Beyond. *Alcoholism: Clinical and Experimental Research* 27(2):232–243, 2003. NOTES: ACTH = adrenocorticotrophin; AMYG = amygdala; BNST = bed nucleus of the stria terminalis; CRF = corticotropin-releasing factor; HPC = hippocampus; NE = nore-pinephine; PFC, prefrontal cortex; PIT = pituitary gland; PVN = paraventricular nucleus.

The hypothalamus, which controls consummatory behavior and basic drives related to food, water, sex, and temperature (Miller 1958), is a complex brain region with reciprocal connections to numerous structures, including the cortex, striatum, hippocampus, amygdala, cerebellum, and thalamus (Afifi and Bergman 1998). The paraventricular nucleus is located in the anterior division of the hypothalamus and includes magnocellular and parvocellular cells. Parvocellular cells are responsible for the release of the stress-associated hormone, cortiocotropin-releasing factor (CRF), which regulates the secretion of the pituitary hormone, adrenocorticotropin. Adrenocorticotropin (also known as ACTH or corticoptropin), in turn, stimulates the adrenal gland to boost the synthesis of corticosteroid hormones (e.g., glucocorticoids such as cortisol and mineralocorticoids such as aldosterone) (Heimer 1995) (figure 6A).

Both acute and chronic alcohol consumption activate the hypothalamic–pituitary–adrenal (HPA) axis, and chronic alcoholism is associated with low basal cortisol and blunted ACTH and cortisol responses to CRF (Adinoff et al. 1990). Disruption of the HPA axis following exposure to alcohol has been demonstrated in rodents (Rasmussen et al. 2000). In the Rhesus Macaque, a single-nucleotide polymorphism in the CRF gene (−2232 C>G) conferred a decreased sensitivity of the CRF promoter to corticosteroid regulation in vitro and was associated with lower levels of CRF in cerebrospinal fluid. Monkeys with this polymorphism tended to be more exploratory and exhibited increased alcohol consumption compared with the monkeys in which this single nucleotide was unmodified (Barr et al. 2008).

The link between the body’s response to stress and alcohol is complex. One theory—the negative reinforcement theory—states that people continue to self-administer alcohol even after the rewarding effects of alcohol are blunted and when alcohol use causes adverse effects on lifestyle (DSM–IV criteria 6) or exacerbates psychological and physiological problems (DSM–IV criteria 7) in order to avoid the negative emotional states (e.g., stress) associated with withdrawal (see the article by Gilpin and Koob, pp. 185–195). The mechanisms behind this negative reinforcement are believed to involve an extensive extrahypothalamic CRF system centered on the extended amygdala (figure 6B).

Evidence for increased CRF activity in the extended amygdala, which may contribute to excessive alcohol consumption, has come from alcohol-dependent rats (i.e., rats exposed to alcohol vapor for 4 weeks, during which BALs reached ∼200 mg/dl). Dependent animals display a significant increase in self-administration of alcohol compared with baseline self-administration (Valdez et al. 2002). Injections of the CRF antagonist d-phe-CRF_12–41_ into the central nucleus of the amygdala, but not the lateral bed nucleus of the stria terminalis or nucleus accumbens shell (figure 6C) in alcohol-dependent animals, reduced alcohol self-administration (Funk et al. 2006). Because blocking CRF receptors reduced alcohol consumption, these results support the view that CRF in the central nucleus of the amygdala plays a role in mediating excessive alcohol consumption in dependent animals.

Although no studies to date have used dogs to explore the stress theory of alcohol abuse, the nervous pointer dog is a candidate for future research. Not all pointer dogs are nervous, but in those animals in which anxious behavior is noted, catecholamine alterations occur (Gurguis et al. 1990). Recent evidence has found that brainstem catecholamines, some of which are activated by stressors, may mediate HPA axis hyperactivity in alcoholism (Choi et al. 2008).

#### Limitations of Animal Models of Stress

CRF initializes the synthesis of corticosteroid hormones, which, in turn, act on glucocorticoid receptors in the brain. Glucocorticoid receptors act as nuclear transcription factors and contribute to the regulation of brain cell properties by modifying the transcription of responsive genes, and therefore, protein synthesis (de Kloet et al. 2005).

In adulthood there is high consistency across animal species in terms of the brain regions that express glucocorticoid receptors, although the levels of expression can differ (e.g., rodents exhibit relatively high glucocorticoid receptor expression in the CA1-2 subfields of the hippocampus and primates exhibit relatively high glucocorticoid receptor expression in the neocortex) (Pryce 2008). Significantly, the relative densities of these receptors differ considerably during postnatal development, creating species-specific periods of critical vulnerability. For example, early life stress in a species that exhibits low glucocorticoid receptor expression in infancy could be less harmful than early life stress in a species that exhibits high glucocorticoid receptor expression, because there are fewer receptors to mediate the effects of elevated cortisol (Fuchs and Flugge 2002). These findings are relevant when modeling alcoholism in animals, especially in light of evidence that the onset of stress-related disorders is age dependent.

### Inflammation

Inflammatory responses to alcohol may contribute to alcohol-related brain damage. Systemic cytokines (i.e., signalling proteins used extensively in cellular communication), particularly tumor necrosis factor-α (TNFα), may enter the brain to initiate inflammatory processes (Qin et al. 2007). The brain’s immune defense cells (i.e., microglia) respond by activating central proinflammatory cytokines (e.g., interleukin 1β [IL1β] and TNFα), which, in turn, can stimulate microglia to produce monocyte chemoattractant protein 1 (MCP-1, Qin et al. 2007). MCP-1 directly induces programmed cell death (i.e., neuronal apoptosis) (Kalehua et al. 2004). Thus, increased MCP-1 observed in brain tissue (VTA, substantia nigra, hippocampus, and amygdala) from alcoholics relative to control subjects (He and Crews 2008) could directly cause neuronal damage and thus could be one of the mechanisms contributing to alcohol-related neuronal loss and brain atrophy.

Various lines of evidence now support the contention that white matter in the brain is particularly sensitive to the damaging effects of alcohol. MR diffusion tensor imaging (DTI) in humans reveals abnormalities in the white matter subadjacent to frontal cortical regions (i.e., centrum semiovale) and the corpus callosum (Nagel and Kroenke, pp. 242–246; see also the article by Rosenbloom and Pfefferbaum, Part 2) and implicates deficits in both myelination and axonal integrity (Pfefferbaum et al. 2000, 2006*b*,*c*; Pfefferbaum and Sullivan 2002, 2005). Postmortem studies of brains of human alcoholics support the finding that white matter is especially affected (Badsberg-Jensen and Pakkenberg 1993; De la Monte 1988; Harper and Kril 1991, 1994), and volume reductions, demyelination, loss of myelinated fibers, and axonal deletions also have been observed (Alling and Bostrom 1980; Harper et al. 1988; Harper and Kril 1989; Kril et al. 1997).

Consistent with these results are molecular studies of human brains which show that the expression of genes encoding myelin proteins (Lewohl et al. 2000; Mayfield et al. 2002) and the actual levels of myelin-associated proteins are decreased in people with alcoholic relatives compared with control cases without a family history of alcoholism (Hasin et al. 2006; Lewohl et al. 2005).

In dogs exposed to alcohol for 1 year, fewer glial cells were found in the temporal and frontal cortices compared with control animals (Hansen et al. 1991), suggesting a reduced capacity for myelin generation. In rats longitudinally exposed to alcohol, in vivo MRI revealed that alcohol significantly slowed corpus callosum growth compared with control animals (Pfefferbaum et al. 2006*a*), and postmortem analysis suggests that the corpus callosum is significantly thinner in the alcohol-exposed group compared with the control group (He et al. 2007).

In light of the evidence indicating that brain white matter is especially vulnerable to the damaging effects of alcohol, neuroinflammation appears to be a likely mechanism of harm to this constituent of the brain. MCP-1 is associated with demyelination in a variety of experimental animal models (Kim and Perlman 2005), and microglia can cause white matter damage via excitotoxicity (i.e., they can impair glutamate uptake by reducing the expression of glutamate transporters) (Matute et al. 2006, 2007).

Inflammation in the adult hippocampus may interfere with memory by inhibiting neurogenesis (Das and Basu 2008). In rats, binge-like exposure to alcohol is marked by local neuroinflammation, which inhibits hippocampal neurogenesis (Nixon and Crews 2002). Increases in TNFα and MCP-1 mRNA levels were observed in male C57BL/6J mice given alcohol intragastrically for 1 day. Ten daily doses of alcohol significantly elevated both mRNA and protein levels of TNFα and MCP-1; however, neither a single dose nor 10 daily doses of alcohol inhibited neurogenesis in the hippocampus of these mice (Qin et al. 2008). Thus, a causal relationship between alcohol-induced neuroinflammation and alcohol-induced suppression of neurogenesis has yet to be established, and further work is required to demonstrate how prolonged elevations in brain cytokines may contribute to neuropathology.

#### Limitations of Animal Models of Inflammation

The neuroinflammation theory of alcohol-related neuronal loss and brain atrophy is relatively new. As a result, there have been few studies designed to specifically test the hypothesis. With respect to the effects of neuroinflammation on neurogenesis, major differences exist between the rat and mouse stem/progenitor cells that are involved in neurogenesis (Ray and Gage 2006), which warrants caution when drawing inferences from one species to another.

### Evidence for Recovery with Abstinence

From the earliest computed tomography (CT) studies to current MRI studies aimed at tracking evidence for brain structural recovery, there has been positive support for at least partial reversal of brain tissue shrinkage with abstinence from alcohol (CT studies: Cala et al. 1983; Carlen et al. 1984, 1986) (MRI studies: Cardenas et al. 2007; Pfefferbaum et al. 1995, 1998).

Indeed, alcoholic brain pathology can be subsumed under Carlen’s two-component hypothesis (Carlen et al. 1984), one reflecting permanent change (i.e., irreversible neuronal cell death), notably in the superior frontal association cortex, and one reflecting a transient change, such as shrinkage without cell death, thereby permitting volume to change (up or down) without long-term damage. Indeed, the majority of shrinkage with drinking does not necessarily reflect “neuronal loss.” Rather, the controlled longitudinal imaging studies demonstrating volume reductions likely reflect nonneuronal loss and neuronal cell body and process shrinkage. That brain volume can increase and that this increase predicts improvement in neuropsychological test performance (Cardenas et al. 2007; Rosenbloom et al. 2007; Sullivan et al. 2000*b*) supports the contention that little neuronal death occurs with alcoholism.

### Animal Models of Recovery

In aged Fisher 344 rats, recovery after long-term treatment with alcohol was associated with a restoration of the total number of synapses on Purkinje neurons of the cerebellum lost during exposure (Dlugos and Pentney 1997). Furthermore, abstinence for 5 weeks indicated a twofold increase in new neurons formed in neurogenetic zones of abstinent animals compared with alcohol-naive animals (Nixon and Crews 2004). This increase in neurogenesis during abstinence from chronic alcohol exposure may be related to the recovery of brain volume deficits (Pfefferbaum et al. 1995) and cognitive deficits in abstinent alcoholics (Sullivan et al. 2000*c*).

## Conclusion

Together, studies in humans and animal models provide support for the involvement of specific brain structures over the course of alcohol addiction. Researchers have identified genetic variants of key inhibitory receptors in the prefrontal cortex that may produce a heritable vulnerability to alcohol, perhaps accounting for the disinhibited personality type observed in certain alcoholics and which leads to a predisposition to develop alcoholism.

The prefrontal cortex and its complex circuitry with the basal ganglia also is likely involved in the acute reinforcing (or rewarding) effects of alcohol. Furthermore, modified prefrontal inhibitory receptors may contribute to dysregulation in other brain regions targeted by the prefrontal cortex, such as the cerebellum. The basal ganglia, cerebellum, amygdala, and hippocampus may collectively contribute to the formation of an alcohol habit. The HPA axis additionally has a role in the development of dependence, as well as the vulnerability to stress-induced relapse. Inflammatory cascades initiated by chronic alcohol consumption are a factor that may contribute to alcohol-induced neuropathology.

**Figure 7:**
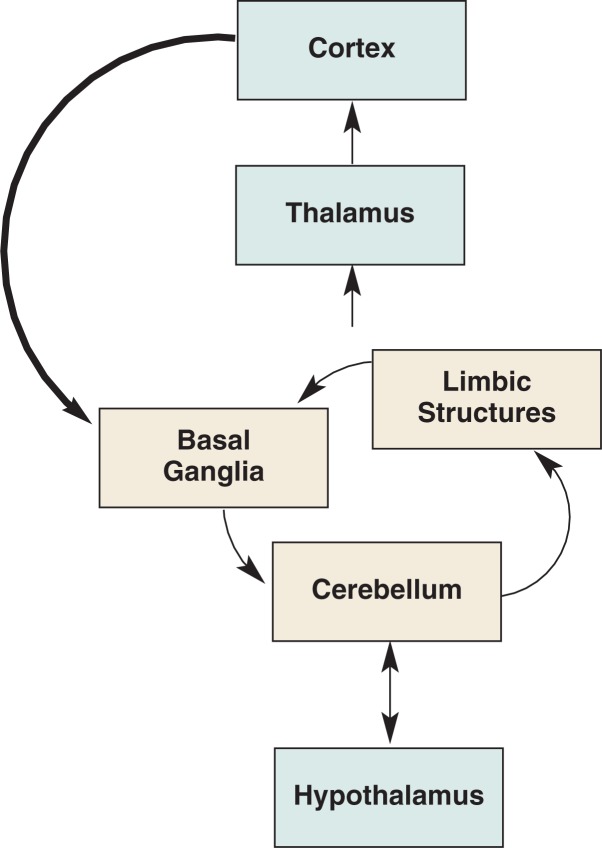
Schematic diagram representing the brain systems modified by alcohol.

Each theory, linked to specific brain structures, has helped to describe the mechanisms associated with the transition from controlled drinking to compulsive consumption or dependence. The development of each theory depended critically on information acquired from animal models, whether they met all the criteria necessary for an animal model of alcoholism or not. Figure 7 is a simplified schematic of the brain structures modified by alcohol and illustrates reciprocal connections between basal ganglia, limbic structures (i.e., hippocampus and amygdala), and cerebellum, each driven by inputs from the cortex, with reciprocal connections to the cortex via the thalamus. Also illustrated are the reciprocal connections between basal ganglia, limbic structures, and cerebellum with the hypothalamus. Not illustrated but germane to the course of alcohol addiction are modifying aminergic (dopamine and norepinephrine), cholinergic, serotonergic, peptidergic, and hormonal influences on the various structures.

In moving forward, a challenge will be to develop a theory that accounts for the brain structures uniquely targeted by alcohol. Perhaps different neural circuits are important at different stages across the time course from first drink to dependence. Alternatively, differential involvement of these circuits across alcoholics could contribute to heterogeneity in brain regions affected. A theory that unifies the brain circuitries modified by alcohol may very well have a major impact on our understanding of brain function in general.

## Figures and Tables

**Figure 1:**
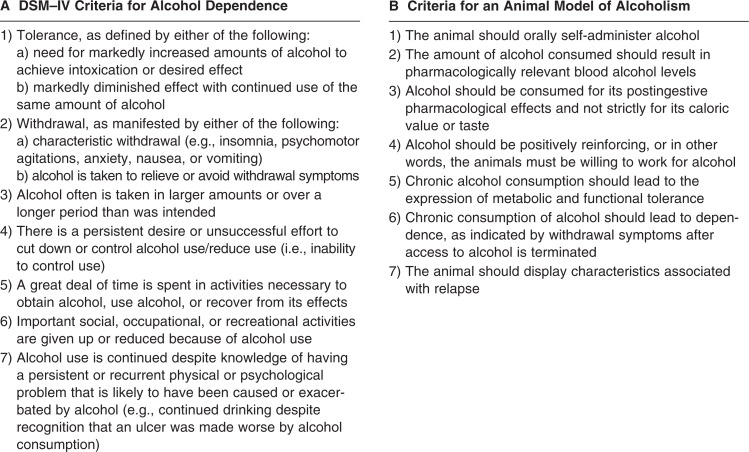
**A)** DSM–IV criteria for alcohol dependence. **B)** Criteria for an animal model of alcoholism.
